# High-flow arteriovenous fistula and myocardial fibrosis in hemodialysis patients with non-contrast cardiac magnetic resonance imaging

**DOI:** 10.3389/fcvm.2022.922593

**Published:** 2022-07-27

**Authors:** Yong Seon Choi, In Jae Lee, Jung Nam An, Young Rim Song, Sung Gyun Kim, Hyung Seok Lee, Jwa-Kyung Kim

**Affiliations:** ^1^Department of Internal Medicine & Kidney Research Institute, Hallym University Sacred Heart Hospital, Anyang, South Korea; ^2^Department of Radiology, Hallym University Sacred Heart Hospital, Anyang, South Korea

**Keywords:** hemodialysis, access flow, cardiac fibrosis, native T1, galectin-3

## Abstract

**Background:**

The role of high-flow arteriovenous fistula (AVF) in cardiovascular morbidity in hemodialysis (HD) patients is very likely under-recognized. We assessed the relationship between high access flow (Qa) and myocardial fibrosis in HD patients.

**Methods:**

Myocardial fibrosis was assessed by native T1 relaxation times on non-contrast cardiac magnetic resonance imaging and a potential marker of fibrosis. Serum levels of galectin-3, N-terminal pro-B-type natriuretic peptide (NT-proBNP), and monocyte chemoattractant protein 1 (MCP-1) were measured in 101 HD patients who underwent regular monitoring of AVF Qa. A high-flow AVF was defined as a Qa >2 L/min.

**Results:**

Hemodialysis patients showed significantly higher galectin-3 value and increased T1 relaxation time compared to healthy volunteers, suggesting increased myocardial fibrosis in uremic cardiomyopathy. In HD patients, 20 (19.8%) had a Qa > 2L/min, and they had significantly higher cardiac output, cardiac index, left ventricular mass, and increased T1 times than those with a Qa ≤ 2 L/min. Also, serum galectin-3 and NT-proBNP levels were much higher in the high Qa group, indicating a close relationship between the high Qa, increased myocardial fibrosis, and the risk of heart failure (HF) in HD patients. It is interesting that a higher AVF Qa for myocardial fibrosis was independent of several traditional cardiovascular risk factors as well as serum levels of NT-proBNP and MCP-1.

**Conclusions:**

A supra-physiologically high Qa can be related to myocardial fibrosis and increased risk of HF in HD patients. Regular Qa monitoring could allow early detection of a high-flow AVF that could arise cardiac complications.

## Introduction

Patients with end-stage renal disease (ESRD) on hemodialysis (HD) have very high mortality, and sudden cardiac death (SCD) is the single most common form of death, accounting for 20% to 30% of all deaths in this population (1, 2). As a significant proportion of SCD events are not caused by ischemic coronary artery disease (CAD) (3), the importance of structural heart diseases, such as left ventricular hypertrophy (LVH) and myocardial fibrosis has been emphasized as a prognostic factor for mortality (4, 5). In particular, myocardial fibrosis is a critical part of maladaptive cardiac remodeling that leads to heart failure (HF) (6). In patients with chronic kidney disease (CKD), diffuse myocardial fibrosis is a typical characteristic of uremic cardiomyopathy unrelated to CAD (7, 8). It progresses with each stage of CKD and is the most severe in HD patients (9–11). Therefore, increased myocardial fibrosis can be a major risk factor for premature death and cardiovascular (CV) diseases in HD patients (7, 9, 12).

The creation of a new functioning arteriovenous fistula (AVF) in HD patients causes an acute decrease in systemic vascular resistance and a secondary increase in cardiac output (CO) (13–15). The increased CO and greater venous return lead to increased left ventricular (LV) pressure and higher LV mass causing LVH (16, 17). In general, these hemodynamic changes are clinically insignificant, however, in certain cases (such as high-flow AVF), they can cause new or worsening heart failure (HF), so-called high-output HF (18, 19). Therefore, the long-standing AVF might be one of the factors aggravating myocardial fibrosis in HD patients, especially if it is a high-flow AVF. To date, AVF with Qa >2 L/min is generally considered as a high-flow AVF, and these patients are at increased risk for developing high-output HF (20). Therefore, the Kidney Disease Outcomes Quality Initiative guideline considers it reasonable to closely monitor Qa every 6–12 months and prophylactically manage high-flow access to avoid serious or irreversible CV complications (21).

As myocardial fibrosis has been proven reversible and treatable only under timely intervention, risk identification and early detection of myocardial fibrosis can be extremely important in HD patients. In this regard, we focused on the association between high intra-access Qa and the extent of myocardial fibrosis in chronic HD patients. For detection of myocardial fibrosis in HD patients, non-contrast cardiac magnetic resonance imaging (MRI) and circulating biomarker have been widely used (16, 22, 23) as the use of gadolinium contrast is challenging in this population given the association between gadolinium and nephrogenic systemic fibrosis (24, 25). Indeed, non-contrast native T1 relaxation time, which is correlated with cardiac fibrosis found on tissue histology, is emerging as a feasible alternative to the use of gadolinium contrast for many patients including those with CKD and HD (12, 23).

In this study, the degree of myocardial fibrosis was assessed with non-contrast cardiac MRI–derived native T1 relaxation time and an emerging myocardial fibrosis biomarker, galectin-3. In addition, levels of the traditional cardiac risk marker, N-terminal (NT)-pro hormone BNP (NT-proBNP), and inflammatory cytokines were measured to examine their association with access Qa and cardiac fibrosis.

## Materials and methods

### Participants

The associations of high intra-access Qa with cardiac parameters and fibrosis were assessed. Patients with an AV graft were not included. In Korea, when AVG placement is needed, a graft of 6 mm diameter is generally used. So, the mean flow of AVG is mostly within 800–1,400 ml/min. So, none of the patients with AVG had an intra-access flow over 2 L/min in our facility. So, a total of 156 patients with AVFs were screened, and 55 were excluded. The exclusion criteria were as follows: history of severe lung disease, such as chronic obstructive pulmonary disease over grade 3 or 4 or asthma or extensive lung cancer (*n* = 5); current or prior symptoms of CHF with moderate or severe valvular heart disease or a decreased ejection fraction <30% (*n* = 9); central venous stenosis (*n* = 3); refusal to measure access flow using Transonic US (*n* = 35); and other (*n* = 3). Ultimately 101 patients were included in the study. Baseline demographic data, including age, sex, comorbidities, and clinical data regarding the underlying cause of renal disease, were obtained. Body mass index was calculated as body weight (kg)/(height/100, m)^2^. Venous sampling was performed immediately prior to each patient's mid-week HD session. Laboratory analyses included white blood cells, hemoglobin, blood urea nitrogen, creatinine, albumin, calcium, and phosphate levels. The study was approved by the Hallym University Sacred Heart Hospital Institutional Review Board and was conducted in accordance with the Declaration of Helsinki. Informed consent was obtained from HD patients and healthy volunteers (HVs).

### Biomarkers

A Serum Separator Clot Activator tube (456073, BD) was used to collect samples from 40 HVs and 101 HD patients. Blood samples were collected at the beginning of dialysis and incubated for 20–30 min at room temperature and then centrifuged for 20 min at 1,000 g. Isolated serum was aliquoted and stored at −70°C until further analysis. We quantified concentrations of NT-proBNP, galectin-3, tumor necrosis factor alpha (TNF-α), and monocyte chemoattractant protein 1 (MCP-1) using ELISA kits (R&D Systems, Minneapolis, MN, USA) according to the manufacturer's instructions.

### Access flow measurements with transonic US

Ultrasound (US) dilution technique (Transonic®) is a widely used non-invasive method to monitor intra-access Qa. This is easy to use and generates accurate measurements [25]. Recently, a more updated form (Transonic HD03 cardiac function monitoring) enables cardiac function screening of CO and the cardiac index (CI) with the concomitant intra-access Qa measurements. In this study, intra-access Qa and cardiac parameters were simultaneously measured with the Transonic HD03CO cardiac monitor (Transonic Systems, Ithaca, NY, USA). Briefly, a 30 ml bolus of saline (saline indicator) was introduced *via* the venous HD line into the access flow stream. After passing through the heart, the US sensor on the arterial HD line recorded the dilution curve resulting from the saline indicator. Then CI was calculated with conventional Stewart-Hamilton analysis. Mean blood pressure (BP) was obtained with an automated BP cuff. In addition, a Qa/CO ratio was obtained by the HD03CO with a reversed blood lines connection and saline injection. Intra-access Qa was measured regularly once a month, and the mean access flow over 6 months was used in this study. Inter-observer and intra-observer variability in Qa was assessed with the intraclass correlation coefficient. The inter-observer and intra-observer variability was 0.95 and 0.92, respectively. Cardiac parameters, including CO, Qa/CO, and CI, were measured twice.

### Cardiac MRI acquisition

Cardiac MRI was performed in 35 of the 101 HD patients and in 14 HVs. Of the patients who did not receive MRI, 32 refused, 25 failed to hold their breath or lie still during the test, and the rest had a contraindication to the MRI test. We obtained multimodal imaging data using a 3-T MRI device (Skyra; Siemens Medical Solutions, Erlangen, Germany). In HD patients, MRI was consistently performed on non-dialysis or post-dialysis days according to the availability of MRI. Left ventricular ejection fraction (LVEF), left ventricular end-systolic volume (LV ESV), left ventricular end-diastolic volume (LV EDV), CO, stroke volume (SV), left ventricular mass (LVM), and LVM index (LVMI) were automatically calculated and indexed to body surface area with a weight acquired immediately prior to scan.

### Image analysis

Two blind observers analyzed anonymized images in random order to determine T1 relaxation time. From the raw T1 images, LV contours were defined and copied onto color-enhanced spatially co-registered maps. Using the anterior right ventricular-LV insertion post as a reference, T1 maps were segmented according to the American Heart Association's 16-segment model. Regions of interest were delineated by user-defined semi-automated border delineation (Syngo *via* workstation) and standardized to a similar size and shape. After the removal of any segments affected by artifacts, a global T1 time was calculated from the mean of all remaining segments. We calculated a septal T1 time by averaging the acceptable anteroseptal, inferoseptal, and septal segments (segments 2, 3, 8, 9, and 14). Mid-septal T1 time was derived from an average of included septal segments from the midshort-axis slice (segments 8 and 9). The reproducibility of the T1 map was assessed by blind reanalyzes of 20 randomly selected images by the same observer. Ten participants were selected at random to determine intra-observer reproducibility, and the correlation coefficients were 0.86–0.96 for all parameters, indicating excellent agreement.

### Statistical analysis

Variables with normal distributions were expressed as means ± standard deviations. Categorical variables were expressed as percentages and compared using the chi-square test. Correlations between continuous variables were tested with either the Pearson's *R* or Spearman's rank correlation coefficient, as appropriate. We tested the association between the access Qa and degree of myocardial fibrosis using native T1 relaxation time and serum galectin in multivariate linear regression models. Model 1 was not adjusted, Model 2 incorporated age and sex, Model 3 was further adjusted for risk factors for structural heart disease, and Model 4 was adjusted for NT-proBNP and MCP-1 levels. To show the predictive value of Qa >2 L/min for myocardial fibrosis, the highest quartile (Q4) level of galectin-3 was determined as the cutoff value for myocardial fibrosis. All statistical analyses were performed with SPSS (version 20.0; IBM, Chicago, IL, USA), and *p* <0.05 was accepted as significant.

## Results

### Participants

**Figure 1:**
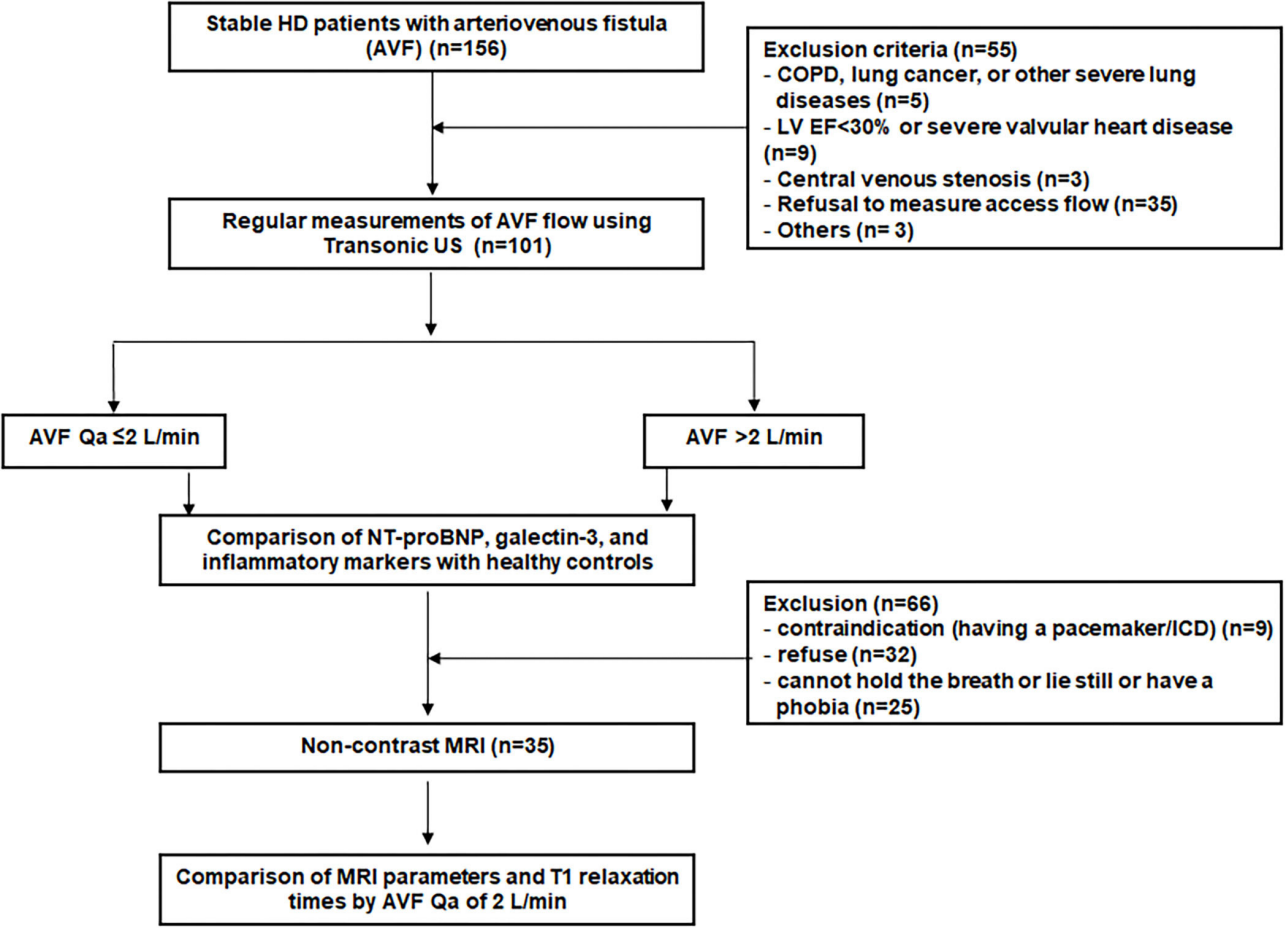
Flow chart of the patient enrollment.

A total of 101 stable HD patients with AVFs and 40 age- and sex-matched HVs were included. Flow chart of patient inclusion are shown in Figure 1. The mean age of the participants was 66.8 ± 11.9 years, and 58.4% of patients were male. A total of 57.4% of patients had upper arm AVFs, in which the mean Qa was 1,750 ± 683 ml/min. In the 42.6% of patients with lower arm AVFs, the mean Qa was 1,233 ± 563 ml/min. Consistent with the general consensus, a Qa >2 L/min was regarded as a cutoff indicator of a high-flow AVF, and 20 (19.8%) patients had a high-flow AVF in this study.

Baseline demographics, biochemical characteristics, and CV risk factors were compared according to a mean Qa of 2 L/min (Table 1). Most of the patients (85%) with a high Qa had an upper arm AVF. Prevalence of CAD, dialysis vintage, levels of biochemical parameters, and medication history were similar between the two groups. Cardiac parameters, however, differed significantly by AVF Qa. Transonic-measured CO was much higher in patients a high Qa compared to those with a low Qa (6.9 vs. 5.0, *p* < 0.001). Also, the mean Qa/CO and CI were significantly increased in this group, too (0.35 ± 0.10 and 4.3 ± 0.7 in patients with Qa >2 L/min, and 0.25 ± 0.08 and 3.0 ± 0.6 in patients with Qa ≤ 2 L/min, respectively).

**Table 1:** Baseline clinical and biochemical data according to the intra-access flow.

**Variables**	**Access flow measurement**				
	**Total**	≤ **2 L/min**	>**2 L/min**	***p*** **-Value**	**Cardiac MRI**
	**(*****n*** = **101)**	**(*****n*** = **81, 80.2%)**	**(*****n*** = **20, 19.8%)**		**(*****n*** = **35)**
Age (years)	66.8 ± 11.9	67.1 ± 11.3	65.4 ± 11.9	0.572	66.0 ± 13.8
Sex, male, *n* (%)	59 (58.4)	49 (60.5)	10 (50.0)	0.216	18 (51.4)
Dialysis Vintage, median (IQR)*	38.6 (28, 58)	37.1 (28, 57)	43.2 (27, 85)	0.221	44.6 (26. 66)
Time from AVF creation, median (IQR)*	45.6 (35, 72)	45.2 (35, 70)	49.6 (39, 82)	0.169	46.6 (27, 70)
**AVF location**					
Upper arm	58 (57.4)	41 (50.6)	17 (85.0)	0.006	25 (71.4)
Lower arm	43 (42.6)	40 (49.4)	3 (15.0)		10 (28.6)
SBP (mmHg)	139.7 ± 17.4	141.0 ± 17.1	134.1 ± 18.1	0.120	140.8 ± 18.3
DBP (mmHg)	72.5 ± 10.0	72.6 ± 9.5	71.7 ± 12.1	0.731	73.5 ± 10.4
BMI (kg/m^2^)	23.2 ± 3.8	23.3 ± 3.9	22.8 ± 3.6	0.658	23.4 ± 3.4
Previous CV events, *n* (%)	20 (19.8)	15 (18.3)	5 (25.0)	0.877	6 (20.0)
DM, *n* (%)	56 (55.4)	50 (61.7)	6 (30.0)	0.010	17 (48.5)
**Laboratory parameters**					
WBC (/μl)	5,890 ± 1,618	5,930 ± 1,630	5,754 ± 1,732	0.667	6,084 ± 1,599
Hemoglobin (g/dl)	10.8 ± 1.0	10.8 ± 0.9	10.7 ± 1.2	0.528	10.9 ± 1.0
BUN (mg/dl)	54.9 ± 15.8	53.7 ± 14.3	59.9 ± 20.7	0.155	57.5 ± 16.9
Creatinine (mg/dl)	8.9 ± 2.3	8.9 ± 2.2	9.0 ± 2.8	0.811	8.9 ± 2.3
Albumin (g/dl)	3.88 ± 0.41	3.90 ± 0.43	3.79 ± 0.34	0.458	3.9 ± 0.3
Ca (mg/dl)	8.7 ± 0.6	8.7 ± 0.6	8.6 ± 0.5	0.455	8.7 ± 0.5
P (mg/dl)	4.9 ± 1.3	5.0 ± 1.4	4.8 ± 1.0	0.618	5.0 ± 1.2
Medications
RAS blocker	64 (63.4)	52 (64.2)	12 (60.0)	0.681	22 (62.8)
Beta-blocker	46 (45.5)	35 (43.2)	11 (55.0)		20 (57.0)
Diuretics	36 (35.6)	28 (34.5)	8 (40.0)		12 (34.1)
Statin	71 (70.3)	55 (67.9)	16 (80.0)		23 (65.7)
**Transonic^®^ monitoring**					
Qa, ml/min	1,627.9 ± 716.2	1,278.6 ± 519.4	2,395.3 ± 543.0	<0.001	1,677.0 ± 831.4
Cardiac Ouput (CO)	5.6 ± 1.4	5.0 ± 1.0	6.9 ± 1.5	<0.001	5.8 ± 1.7
Qa/CO ratio	0.28 ± 0.09	0.25 ± 0.08	0.35 ± 0.10	<0.001	0.28 ± 0.1
Cardiac Index (CI)	3.6 ± 0.9	3.0 ± 0.6	4.3 ± 0.7	<0.001	3.6 ± 0.9

All data are expressed as mean ± SD except for those with *, which are expressed as median with range.

### Differences in cardiac MRI parameters according to access Qa

Cardiac MRI was performed in 35 of the 101 HD patients and 14 of the 40 HVs. Baseline characteristics of 35 patients who underwent MRI studies were comparable compared with the entire patients (Table 1).

As shown in Table 2, HD patients had significantly greater CO, CI, LVM, LVMI, SV, and SVI compared to HV controls. CO and LVMI were 5.7 and 80.3 g/m^2^ in the HD group vs. 4.6 and 55.6 g/m^2^ in the HV group, respectively (*p* = 0.024 for CO, *p* = 0.023 for LVMI). However, differences between the two groups in LVEF, LV EDV, LV EDVI, ESV, and LV ESVI did not reach statistical significance.

**Table 2:** Comparison of CMR parameters between patients with a Qa >2 vs. ≤ 2 L/min.

		**AVF Flow, HD patients**			
	**HV**	**Total**	≤ **2 L/min**	>**2 L/min**	***p*** **-Value**
	**(*****n*** = **14)**	**(*****n*** = **35)**	**(*****n*** = **22)**	**(*****n*** = **13)**	
**CMR imaging**					
Ejection fraction (EF, %)	61.8 ± 6.3	61.7 ± 9.6	63.1 ± 9.2	58.1 ± 8.6	0.151
End-diastolic volume (EDV, ml)	113.6 ± 31.4	130.5 ± 28.1	114.0 ± 25.2	155.2 ± 33.6	0.001
End-diastolic volume index (EDVI)	60.0 ± 13.8	71.5 ± 16.1	59.8 ± 13.8	86.5 ± 20.8	<0.001
End-systolic volume (ESV, ml)	44.3 ± 19.5	52.6 ± 24.8	42.6 ± 18.9	68.3 ± 25.6	0.003
End- systolic volume index (ESVI)	23.8 ± 9.4	28.0 ± 13.5	22.4 ± 9.0	37.3 ± 15.0	0.002
Stroke volume (ml)	68.2 ± 12.3*	77.2 ± 14.0	70.7 ± 11.8	87.6 ± 11.0	<0.001
Stroke volume Index (SVI)	37.1 ± 6.2*	41.6 ± 9.0	37.0 ± 7.8	48.5 ± 6.0	<0.001
Cardiac output (CO, L/min)	4.6 ± 1.1*	5.7 ± 0.9	5.3 ± 0.9	6.3 ± 0.5	0.003
Heart rate (HR, /min)	68.3 ± 8.3	74.5 ± 9.5	75.9 ± 9.0	72.3 ± 10.1	0.310
Cardiac Index (CI)	2.6 ± 0.5*	3.5 ± 0.6	3.2 ± 0.5	3.9 ± 0.4	0.006
LV mass (LVM, g)	105.1 ± 28.8*	147.8 ± 32.6	137.2 ± 33.0	161.9 ± 26.7	0.039
LVM index (LVMI)	55.6 ± 13.4*	80.3 ± 20.4	71.3 ± 15.0	93.2 ± 21.6	0.003
**T1 time (ms)**					
Global, mean/SD	1,192.2/25.5*	1,274.0/68.1	1,250.4 /26.9	1,319.5/28.3	0.001
Septal, mean/SD	1,195.9/20.4*	1,283.6/58.7	1,260.6/22.1	1,328.0/22.0	<0.001
Mid-septal, mean/SD	1,188.6/21.1*	1,271/65.1	1,249.6/20.3	1,313.0/23.1	0.009

*p < 0.05 between HV and HD patients.

Among the HD patients, noticeable differences were observed between patients with Qa >2 L/min and Qa ≤ 2 L/min in all CMR-derived cardiac parameters; LV EDV, ESV, SV, CO, CI, LVM, and LVMI were much greater in patients with a high Qa (Table 2). This suggests that high-flow AVFs are closely linked to the changes in cardiac structure. However, even in this case, there were no significant differences in LVEF between the two groups, which suggests that preserved EF cannot be used to rule out cardiac damage caused by a high access Qa. Indeed, a high Qa was significantly associated with increased CO, CI, SV, LV EDV, and LVMI but was not associated with systemic BP or LVEF. As shown in the Table 3, CO and CI measured by Transonic US showed good correlations with those parameters measured by MRI (CO; *R* = 0.568, *p* = 0.001, CI; *R* = 0.500, *p* = 0.005). In addition, transonic US–derived Qa correlated very well with MRI-derived CO and CI as well as LV EDV, SV, and LVMI (Table 3).

**Table 3:** Correlation between CMR imaging indexes, AVF flow and biomarkers.

**Pearson's** ***R***	**US**	**MRI parameters**								**Biomarkers**	
	**Qa**	**EF**	**LVEDV**	**SV**	**CO**	**CI**	**LVMI**	**T1-global**	**T1-septal**	**NT-proBNP**	**Galectin-3**
Age	0.044	0.110	−0.075	0.010	0.094	0.251	−0.127	−0.117	−0.198	−0.080	0.160
Sex	−0.115	0.054	0.159	0.208	0.039	0.281	−0.123	0.401*	0.330*	0.063	−0.037
**Transonic^®^ US of vascular access**											
Qa	–	−0.044	0.480*	0.517*	0.594**	0.511*	0.420*	0.375*	0.421*	0.263*	0.404**
CO	0.681**	−0.219	0.659**	0.673**	0.568*	0.548*	0.608**	0.144	0.242	0.471*	0.276
Qa/CO	0.599**	−0.037	0.065	0.052	0.331	0.408*	−0.031	0.320*	0.254	0.082	0.152
CI	0.698**	−0.289	0.679**	0.708**	0.560*	0.500*	0.411*	0.275	0.317*	0.567**	0.334
**MRI parameters**											
CO	0.594**	0.014	0.502*	0.722**	–	0.810**	0.548*	0.027	0.130	0.244	−0.051
LVMI	0.420*	−0.174	0.666**	0.701**	0.548*	0.405*	–	0.367*	0.355*	0.339	0.249
T1 relaxation time, global	0.375*	−0.158	0.311	0.179	0.027	0.100	0.367*	–	0.937**	0.042	0.378*
**Biomarkers**											
NT-proBNP	0.263*	−0.285	0.408*	0.332	0.244	0.260	0.339	0.042	0.044	–	0.300*
Galectin-3	0.404**	−0.296	0.258	0.051	−0.051	−0.055	0.249	0.378*	0.421*	0.300*	–
TNF-alpha	−0.227*	−0.078	0.172	0.108	0.094	0.245	0.171	0.375*	0.282	0.041	0.035
MCP-1	0.049	−0.255	0.154	−0.063	−0.152	−0.093	0.050	0.320*	0.323*	0.021	0.114

* <0.05.

** <0.001.

### Native T1 times and correlations with other parameters

**Figure 2:**
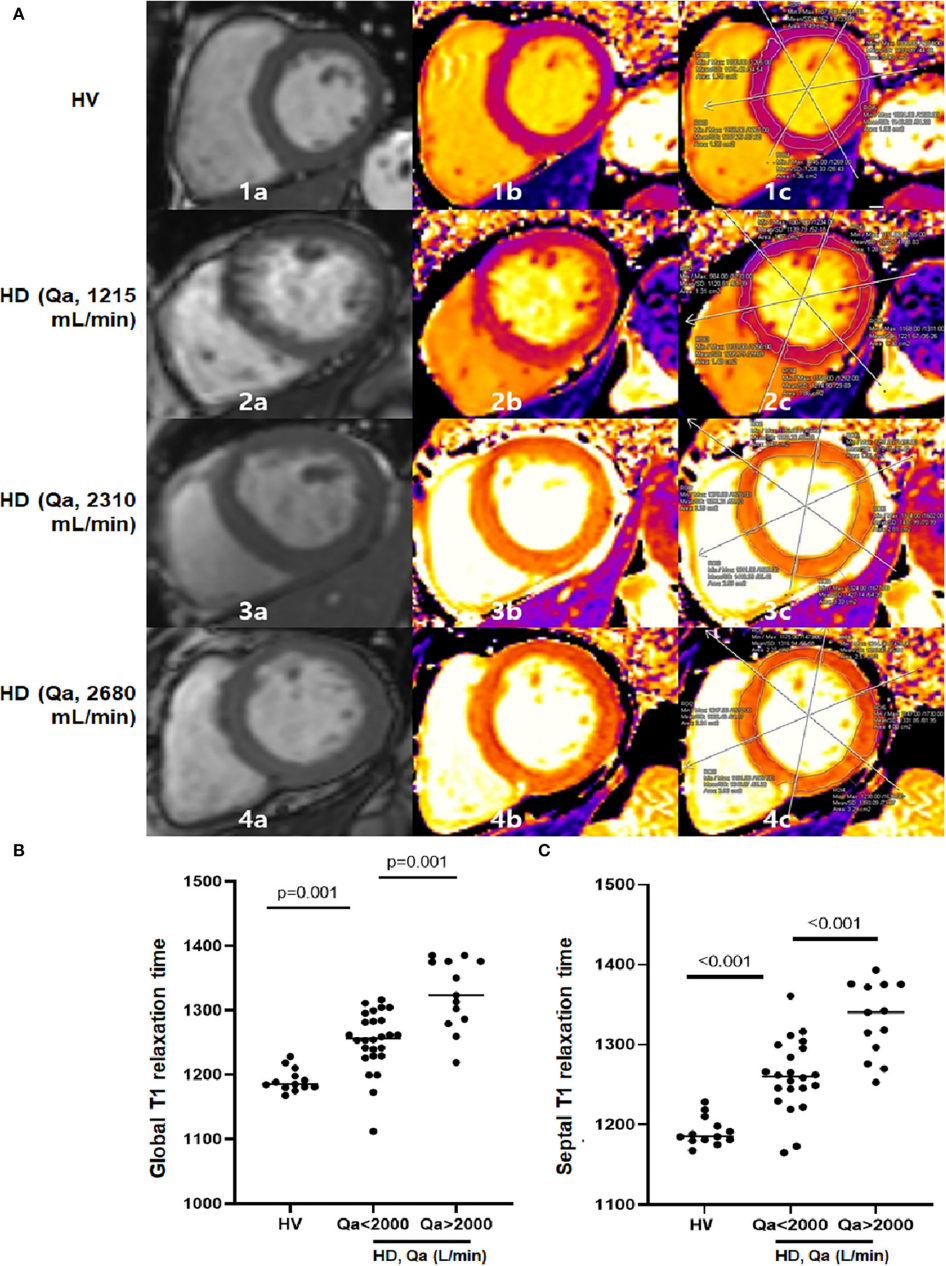
**(A)** Examples of end-diastolic cine images (a), corresponding the native T1 parametric maps (b), and segmented T1 maps (c) in 1 HV and 3 HD patients at the basal level. The figure illustrates how native T1 mapping may be able to detect diffuse interstitial fibrosis. As T1 times increase, color coding changes from purple to orange to yellow. Parts labeled with 1 are HV with mean global T1 of 1,162 ms. Parts labeled with 2 show a HD patient with a Qa of 1,215 ml/min. Mean global T1 is significantly increased at 1,208 ms with a septal T1 of 1,221 ms compared to HV. Parts labeled with 3 and 4 are HD patients with Qa of 2,310 ml/min and 2,680 ml/min. Mean global T1 further increased at 1,327 ms and 1,375 ms, with septal T1 of 1,340 ms and 1,380 ms. **(B,C)** There is a discrete differentiation in global and septal T1 times among HV, HD with a low Qa and HD with a high Qa.

In addition to the MRI -derived cardiac parameters, native T1 relaxation times were used to detect myocardial fibrosis. Representative cine and T1 images as well as T1 regions of interest of HV, HD patients with a low Qa and a high Qa were shown in Figure 2A. Native global, septal, and mid-septal T1 times were much greater in the HD group compared to the HV group, which suggests higher myocardial fibrosis under uremic conditions (*p* <0.001; Table 2). In addition to the discretely increased signal in the HD group, there was a significant difference in native T1 times between patients with Qa >2 L/min and those with Qa <2 L/min (global T1 time: 1,319.5 vs. 1,250.4 ms, *p* = 0.001; septal T1 time: 1,328.0 vs. 1,260.6 ms, *p* <0.001; mid-septal T1 time: 1,313.0 vs. 1,249.6 ms, *p* = 0.009; Figures 2B,C). Also, a close relationship between T1 relaxation time and access Qa was observed in the correlation analysis; Pearson's *R* for global and septal T1 and Qa was 0.375 (*p* = 0.016) and 0.421 (*p* = 0.006). All these findings could indicate that a high intra-access Qa may account for the absolute difference in native T1 values in uremic cardiomyopathy. Moreover, native T1 relaxation times showed a close link with LVMI, indicating the close relationship between the high-flow AVF, increased cardiac mass, and advanced myocardial fibrosis (Table 3).

### Relationships between findings on cardiac MRI and NT-ProBNP, galectin-3, and inflammatory cytokines

**Figure 3:**
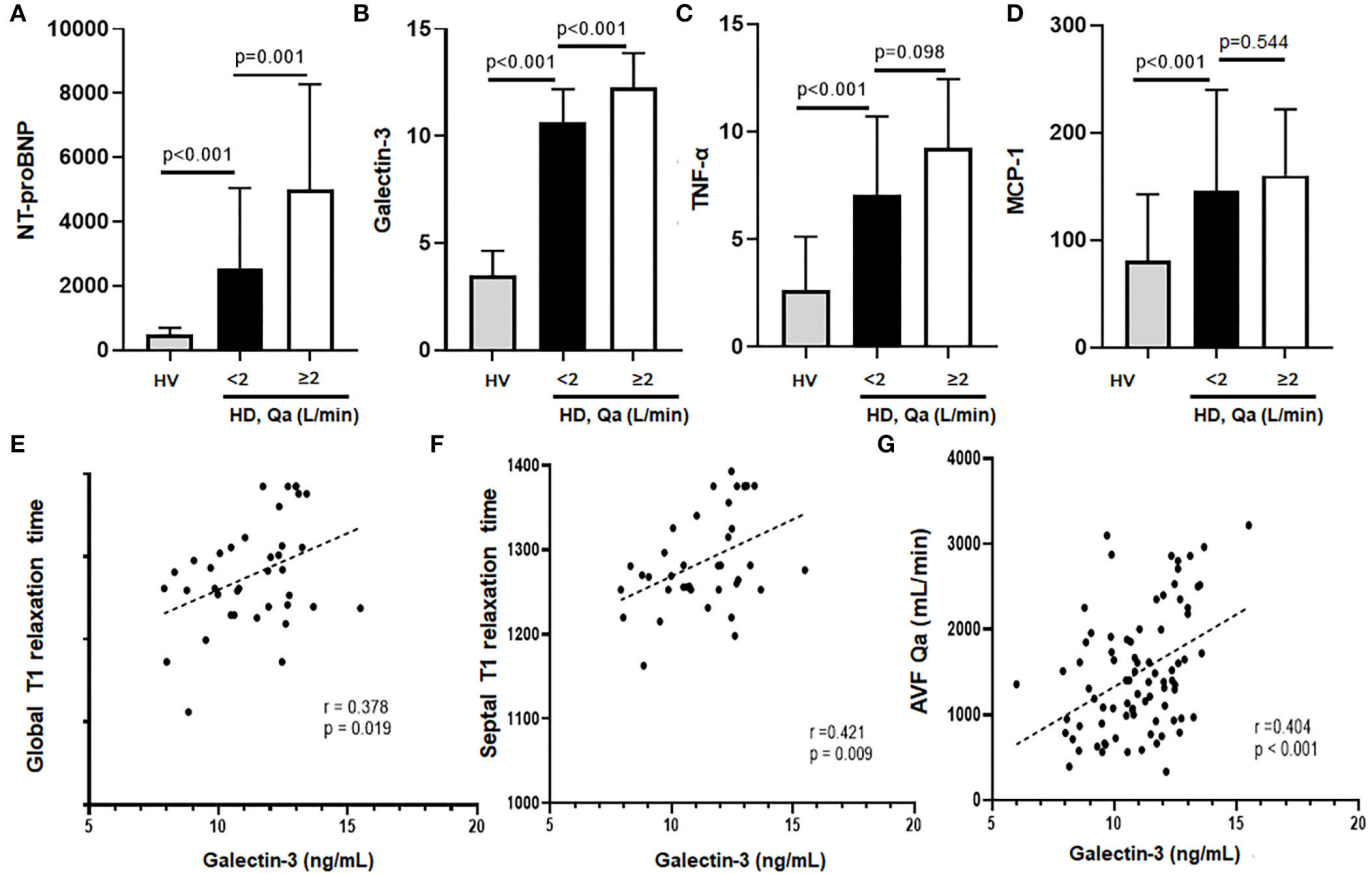
**(A–D)** NT-proBNP, galectin-3, TNF-α, and MCP-1 levels of HD patients vs. HV. All the markers were significantly higher in HD patients compared to HV. In addition, HD patients with a high Qa showed a much higher levels of NT-proBNP and galectin-3 levels compared to HD patients with a low Qa. **(E–G)** Galectin-3 had a close association with global and septal T1 relaxation times as well as mean AVF Qa.

To quantify the myocardial damage, serum levels of biomarkers of HF (NT-proBNP), myocardial fibrosis (galectin-3) and two proinflammatory cytokines (TNF-α and MCP-1) were measured in 101 HD patients and 40 HVs. As expected, serum levels of all four markers were significantly higher in the HD group compared to the HV group (Figures 3A–D).

In the HD group, patients with Qa >2 L/min had significantly higher NT-proBNP and galectin-3 compared to those with Qa <2 L/min (NT-proBNP: 5,009 ± 3,273 vs. 2,549 ± 1,497, galectin-3: 12.27 ± 1.6 vs. 10.6 ± 1.5, respectively; Figures 3A,B). Serum galectin-3 levels correlated very well with native T1 relaxation times, both global (*r* = 0.378, *p* = 0.019) and septal T1 values (*r* = 0.421, *p* = 0.009), suggesting the usefulness of galectin-3 for estimating myocardial fibrosis (Figures 3E,F). Also, we found that galectin-3 levels correlated well with NT-proBNP (*r* = 0.300, *p* = 0.012), and the intra-access Qa (*r* = 0.404, *p* <0.001; Figure 3G; Table 3). All these findings could suggest the increased risk of myocardial fibrosis and HF with a higher Qa. However, levels of TNF-α and MCP-1 did not differ significantly between the two groups. Indeed, although native T1 times was positively associated with MCP-1 and TNF-α, no relationship was observed between galectin-3 and these inflammatory mediators, which suggests an inflammation-independent association of galectin-3 in predicting myocardial fibrosis.

### Factors affecting myocardial fibrosis

To examine the independent relationship between a high intra-access Qa over 2 L/min and the degree of myocardial fibrosis, we performed stepwise multiple regression analyses. As shown in Table 4, a higher Qa >2 L/min was a significant determinant of higher native T1 values and galectin-3 in univariate analyses (Model 1). After adjustments for age, sex, dialysis vintage (Model 2), and traditional risk factors for cardiac disease (Model 3), a higher Qa was a significant contributing factor to increased myocardial fibrosis. Even after we adjusted for NT-proBNP and MCP-1 (Model 4), the close relationship between a high Qa and native T1 value and galectin-3 was consistently observed (global native T1 time: β = 0.627, *p* = 0.005, galectin-3: β = 0.318, *p* = 0.022). Overall, the close relationships between a higher AVF access Qa and myocardial fibrosis might be independent of several traditional risk factors as well as serum levels of NT-proBNP and MCP-1.

**Table 4:** Adjusted association between a high flow AVF and the risk of myocardial fibrosis in HD patients.

	**Model 1**		**Model 2**		**Model 3**		**Model 4**	
	β	***p*** **-Value**	β	***p*** **-Value**	β	***p*** **-Value**	β	***p*** **-Value**
**T1 relaxation time, global**								
Qa >2 L/min	0.486	0.001	0.325	0.026	0.406	0.007	0.627	0.005
**T1 relaxation time, septal**								
Qa >2 L/min	0.552	<0.001	0.410	0.006	0.508	0.002	0.896	<0.001
**Galectin-3**								
Qa >2 L/min	0.403	<0.001	0.428	<0.001	0.404	0.001	0.318	0.022

β, standardized regression coefficient. Model 1, Unadjusted, Model 2, model 1 + age, sex and dialysis vintage, Model 3, model 2 + adjustment for systolic BP, diabetes, and hemoglobin, Model 4, model 3 + adjustment for NT-proBNP and MCP-1.

With a galectin-3 cutoff value of 12.3 ng/ml (the highest quartile of galectin-3) for detecting the presence of myocardial fibrosis, a Qa >2 L/min increased the risk for myocardial fibrosis by 9.6-fold with this cutoff value (odds ratio = 2.97–31.23, *p* < 0.001).

## Discussion

Given the increasing number of HD patients with a very high AVF Qa, the effects of access flow on CV impairment have become an important issue. However, there has been no strong evidence to make a precise definition of parameters that determine high-flow access and high-output HF. Even more, there are no guidelines for when to intervene with patients with a higher Qa and high-output HF. Certainly, the potentially harmful effects of high-flow AVFs on CV morbidities have very likely been under-recognized and overlooked. In this study, the associations of high-flow AVF on myocardial fibrosis were evaluated. To the best of our knowledge, this is the first study to evaluate the relationship between high-flow AVF and increased risk of cardiac fibrosis in HD patients.

Structural heart disease such as increased LVM or cardiac fibrosis appear to be an important cause of increased morbidity and mortality in HD population, but EF monitoring is not sufficient for detecting these changes (1, 17, 23, 26). Myocardial fibrosis is an important part of cardiac remodeling that leads to HF, in particular HF with a preserved EF (HFpEF). HFpEF is a very common clinical syndrome with high morbidity and mortality, and previous data suggest that focal or diffuse myocardial fibrosis may contribute to the pathophysiology of HFpEF (27, 28). Non-traditional risk factors such as chronic inflammation, oxidative stress, volume overload, as well as pre-existing cardiac diseases can induce myocardial fibrosis. Also, obesity-related pulmonary hypertension could cause RV failure as well as HF in ESRD patients (29).

First, in our study, a high-flow AVF was defined with a cutoff value of 2 L/min Qa and we found that HD patients with Qa >2 L/min were more likely to have a higher CO and CI compared to those with Qa ≤ 2 L/min. In addition, those patients had significantly increased LV volume and mass with higher NT-proBNP levels, suggesting high risk of HF. A 2 L/min Qa cutoff value can offer 89% sensitivity and 100% specificity for detecting high-output HF in chronic HD patients (20). Consistent with our finding, Stoumpos et al. (30) recently demonstrated that a newly created AVF resulted in a significant increase in LVM, dimensions of the cardiac chamber, and NT-proBNP levels within weeks after surgery, and these changes were proportional to the fistula flow.

Then, we demonstrate that a high-flow AVF can be a significant determinant of myocardial fibrosis, which is shown as a discretely increased native T1 value coupled with an increase in the validated biomarker galectin-3. Cardiac MRI-derived non-contrast native T1 relaxation time is an emerging marker of cardiac fibrosis in a wide variety of disease states, including CKD and HD. As shown in this study, native T1 values were significantly higher in HD patients compared to HVs. This suggests that diffuse myocardial fibrosis is a typical characteristic of uremic cardiomyopathy (10, 23). Indeed, it is well-known that the late gadolinium enhancement is much more prevalent in uremic cardiomyopathy in the HD population compared to HVs, and its extent is related to hypertrophied, dysfunctional LV segments (31). However, the use of gadolinium in MRI is limited. Thus, native T1 times have recently been considered a useful marker for detecting myocardial fibrosis. Based on these findings, it can be assumed that if HD patients are exposed to a prolonged high CO caused by a high Qa, this can aggravate the degree of myocardial fibrosis with a concomitant increase in LVM. This might compromise the perfusion reserve, which can cause subclinical ischemia and altered regional conduction. As a result, it can increase the risk for fatal arrhythmia and SCD in this population. Consistent with our data, previous study with incident HD patients also reported that global, septal and mid-septal T1 times were all significantly higher in the HD group compared to HV, and the patients showed significantly higher LVMI, too (12).

Also, we found that an increased AVF Qa was also associated with increased markers of cardiac dysfunction, including NT-proBNP and galectin-3. Previous clinical studies have documented marked elevations of circulating galectin-3 concentrations in patients with HF and showed that these levels can predict mortality and adverse outcomes, which suggests a role for galectin-3 as a surrogate biomarker for prognosis (32, 33). Supporting these findings, extensive *in vitro* and *in vivo* evidence associates galectin-3 with a profibrotic effect. The data presented here show that galectin-3 was much higher in patients with a high Qa >2 L/min compared to those with Qa ≤ 2 L/min, and it was closely related to increased native T1 times. Furthermore, this association was independent of other CV risk factors, such as NT-proBNP and serum MCP-1 levels.

Some limitations of this study include the relatively small number of patients who underwent cardiac MRI analyses and the fact that this was a single-center study. However, with a well-characterized cohort of dialysis patients, we were able to perform multivariate adjustments to assess the significance of a high Qa over any other findings. Another limitation of this study is that this study described the association between high- flow AVF and myocardial fibrosis, but we cannot assume a causal relationship from this. To demonstrate this, the patients need to be followed up for future HF events. Also, there may be some risk factors affecting both high-flow AVF and myocardial fibrosis. And, the Qa/CO ratio seems to be high in patients with a Qa <2L/min. In general, a Qa/CO ratio >0.2–0.3 is suggested as a significant risk of developing high-output HF in patients with Qa >2 L/min (34). The clinical significance of the Qa/CO ratio in patients with low Qa need to be further investigated. Lastly, body composition analyses were not performed to rule out patients with volume overload. This is important because water content can prolong T1 times. However, stable patients undergoing maintenance HD were included, and all patients were euvolemic. There was no correlation between T1 time and UF volume or BP. This study would have been strengthened by adding a control population to compare plasma biomarker levels of NT-proBNP, galectin-3, MCP-1, and TNF-α.

In conclusion, a supra-physiologically high Qa can be a serious risk factor for myocardial fibrosis in HD patients. To identify groups at high risk for developing SCD and high-output HF, it seems reasonable to regularly monitor intra-access Qa with concomitant CO and CI measurements using a Transonic US dilution technique.

## Data availability statement

The original contributions presented in the study are included in the article/supplementary material, further inquiries can be directed to the corresponding authors.

## Ethics statement

The study was approved by the Hallym University Sacred Heart Hospital Institutional Review Board and was conducted in accordance with the Declaration of Helsinki. The patients/participants provided their written informed consent to participate in this study.

## Author contributions

J-KK and HL: study design, data analysis and writing up. YC, J-KK, and JA: all experiment and data analysis. IL: experiments setup. YS and SK: data collection and writing up, and statistics statistical advisory. All authors contributed to the article and approved the submitted version.

## Funding

This research was supported by a National Research Foundation grant funded by the Korean government (2020R1A2C110138611) and the Hallym University Research Fund 2020 (HURF-2020-43).

## Conflict of interest

The authors declare that the research was conducted in the absence of any commercial or financial relationships that could be construed as a potential conflict of interest.

## Publisher's note

All claims expressed in this article are solely those of the authors and do not necessarily represent those of their affiliated organizations, or those of the publisher, the editors and the reviewers. Any product that may be evaluated in this article, or claim that may be made by its manufacturer, is not guaranteed or endorsed by the publisher.

## References

[1] Bleyer AJ Hartman J Brannon PC Reeves-Daniel A Satko SG Russell G. Characteristics of sudden death in hemodialysis patients. Kidney Int. (2006) 69:2268–73. 10.1038/sj.ki.5000446 16672908

[2] Allon M. Evidence-based cardiology in hemodialysis patients. J Am Soc Nephrol. (2013) 24:1934–43. 10.1681/ASN.2013060632 24136920 PMC3839557

[3] Herzog CA Asinger RW Berger AK Charytan DM Díez J Hart RG. Cardiovascular disease in chronic kidney disease. A clinical update from Kidney Disease: Improving Global Outcomes (KDIGO). Kidney Int. (2011) 80:572–86. 10.1038/ki.2011.223 21750584

[4] Amann K Breitbach M Ritz E Mall G. Myocyte/capillary mismatch in the heart of uremic patients. J Am Soc Nephrol. (1998) 9:1018–22. 10.1681/ASN.V961018 9621284

[5] Burton JO Jefferies HJ Selby NM Mcintyre CW. Hemodialysis-induced cardiac injury: determinants and associated outcomes. Clin J Am Soc Nephrol. (2009) 4:914–20. 10.2215/CJN.03900808 19357245 PMC2676185

[6] Assomull RG Prasad SK Lyne J Smith G Burman ED Khan M. Cardiovascular magnetic resonance, fibrosis, and prognosis in dilated cardiomyopathy. J Am Coll Cardiol. (2006) 48:1977–85. 10.1016/j.jacc.2006.07.049 17112987

[7] Mall G Huther W Schneider J Lundin P Ritz E. Diffuse intermyocardiocytic fibrosis in uraemic patients. Nephrol Dial Transplant. (1990) 5:39–44. 10.1093/ndt/5.1.39 2109283

[8] Graham-Brown MP Singh AS Gulsin GS Levelt E Arnold JA Stensel DJ. Defining myocardial fibrosis in haemodialysis patients with non-contrast cardiac magnetic resonance. BMC Cardiovasc Disord. (2018) 18:145. 10.1186/s12872-018-0885-2 30005636 PMC6044074

[9] Aoki J Ikari Y Nakajima H Mori M Sugimoto T Hatori M. Clinical and pathologic characteristics of dilated cardiomyopathy in hemodialysis patients. Kidney Int. (2005) 67:333–40. 10.1111/j.1523-1755.2005.00086.x 15610259

[10] Edwards NC Moody WE Chue CD Ferro CJ Townend JN Steeds RP. Defining the natural history of uremic cardiomyopathy in chronic kidney disease: the role of cardiovascular magnetic resonance. JACC Cardiovasc Imaging. (2014) 7:703–14. 10.1016/j.jcmg.2013.09.025 25034920

[11] Edwards NC Moody WE Yuan M Hayer MK Ferro CJ Townend JN. Diffuse interstitial fibrosis and myocardial dysfunction in early chronic kidney disease. Am J Cardiol. (2015) 115:1311–7. 10.1016/j.amjcard.2015.02.015 25769628

[12] Rutherford E Talle MA Mangion K Bell E Rauhalammi SM Roditi G. Defining myocardial tissue abnormalities in end-stage renal failure with cardiac magnetic resonance imaging using native T1 mapping. Kidney Int. (2016) 90:845–52. 10.1016/j.kint.2016.06.014 27503805 PMC5035134

[13] Iwashima Y Horio T Takami Y Inenaga T Nishikimi T Takishita S. Effects of the creation of arteriovenous fistula for hemodialysis on cardiac function and natriuretic peptide levels in CRF. Am J Kidney Dis. (2002) 40:974–82. 10.1053/ajkd.2002.36329 12407642

[14] Macrae JM Pandeya S Humen DP Krivitski N Lindsay RM. Arteriovenous fistula-associated high-output cardiac failure: a review of mechanisms. Am J Kidney Dis. (2004) 43:e17–22. 10.1053/j.ajkd.2004.01.016 15112194

[15] Korsheed S Eldehni MT John SG Fluck RJ Mcintyre CW. Effects of arteriovenous fistula formation on arterial stiffness and cardiovascular performance and function. Nephrol Dial Transplant. (2011) 26:3296–302. 10.1093/ndt/gfq851 21317408

[16] Reddy YNV Obokata M Dean PG Melenovsky V Nath KA Borlaug BA. Long-term cardiovascular changes following creation of arteriovenous fistula in patients with end stage renal disease. Eur Heart J. (2017) 38:1913–23. 10.1093/eurheartj/ehx045 28329100

[17] Pucchio A Mcintyre C Lok C Moist L. Cardiac implications of upper-arm arteriovenous fistulas: a case series. J Vasc Access. (2022) 11297298211066766. 10.1177/11297298211066766 PMC10631279 34991397

[18] Engelberts I Tordoir JH Boon ES Schreij G. High-output cardiac failure due to excessive shunting in a hemodialysis access fistula: an easily overlooked diagnosis. Am J Nephrol. (1995) 15:323–6. 10.1159/000168857 7573191

[19] Reddy YNV Melenovsky V Redfield MM Nishimura RA Borlaug BA. High-output heart failure: a 15-year experience. J Am Coll Cardiol. (2016) 68:473–82. 10.1016/j.jacc.2016.05.043 27470455

[20] Basile C Lomonte C Vernaglione L Casucci F Antonelli M Losurdo N. The relationship between the flow of arteriovenous fistula and cardiac output in haemodialysis patients. Nephrol Dial Transplant. (2008) 23:282–7. 10.1093/ndt/gfm549 17942475

[21] Lok CE Huber TS Lee T Shenoy S Yevzlin AS Abreo K. KDOQI clinical practice guideline for vascular access: 2019 update. Am J Kidney Dis. (2020) 75:S1–s164. 10.1053/j.ajkd.2019.12.001 32778223

[22] Shah AM Solomon SD. Myocardial deformation imaging: current status and future directions. Circulation. (2012) 125:e244–8. 10.1161/CIRCULATIONAHA.111.086348 22249531

[23] Graham-Brown MP March DS Churchward DR Stensel DJ Singh A Arnold R. Novel cardiac nuclear magnetic resonance method for noninvasive assessment of myocardial fibrosis in hemodialysis patients. Kidney Int. (2016) 90:835–44. 10.1016/j.kint.2016.07.014 27633869

[24] Marckmann P Skov L Rossen K Dupont A Damholt MB Heaf JG. Nephrogenic systemic fibrosis: suspected causative role of gadodiamide used for contrast-enhanced magnetic resonance imaging. J Am Soc Nephrol. (2006) 17:2359–62. 10.1681/ASN.2006060601 16885403

[25] Kribben A Witzke O Hillen U Barkhausen J Daul AE Erbel R. Nephrogenic systemic fibrosis: pathogenesis, diagnosis, and therapy. J Am Coll Cardiol. (2009) 53:1621–8. 10.1016/j.jacc.2008.12.061 19406336

[26] Yamada S Ishii H Takahashi H Aoyama T Morita Y Kasuga H. Prognostic value of reduced left ventricular ejection fraction at start of hemodialysis therapy on cardiovascular and all-cause mortality in end-stage renal disease patients. Clin J Am Soc Nephrol. (2010) 5:1793–8. 10.2215/CJN.00050110 20595691 PMC2974379

[27] Borlaug BA Lam CS Roger VL Rodeheffer RJ Redfield MM. Contractility and ventricular systolic stiffening in hypertensive heart disease insights into the pathogenesis of heart failure with preserved ejection fraction. J Am Coll Cardiol. (2009) 54:410–8. 10.1016/j.jacc.2009.05.013 19628115 PMC2753478

[28] Mohammed SF Hussain S Mirzoyev SA Edwards WD Maleszewski JJ Redfield MM. Coronary microvascular rarefaction and myocardial fibrosis in heart failure with preserved ejection fraction. Circulation. (2015) 131:550–9. 10.1161/CIRCULATIONAHA.114.009625 25552356 PMC4324362

[29] Ren J Wu NN Wang S Sowers JR Zhang Y. Obesity cardiomyopathy: Evidence, mechanisms and therapeutic implications. Physiol Rev. (2021) 101:1745–1807. 10.1152/physrev.00030.2020 33949876 PMC8422427

[30] Stoumpos S Rankin A Hall Barrientos P Mangion K Mcgregor E Thomson PC. Interrogating the haemodynamic effects of haemodialysis arteriovenous fistula on cardiac structure and function. Sci Rep. (2021) 11:18102. 10.1038/s41598-021-97625-5 34518583 PMC8437985

[31] Schietinger BJ Brammer GM Wang H Christopher JM Kwon KW Mangrum AJ. Patterns of late gadolinium enhancement in chronic hemodialysis patients. JACC Cardiovasc Imaging. (2008) 1:450–6. 10.1016/j.jcmg.2008.03.011 19356466 PMC2933143

[32] Van Der Velde AR Gullestad L Ueland T Aukrust P Guo Y Adourian A. Prognostic value of changes in galectin-3 levels over time in patients with heart failure: data from CORONA and COACH. Circ Heart Fail. (2013) 6:219–26. 10.1161/CIRCHEARTFAILURE.112.000129 23395934

[33] Hara A Niwa M Kanayama T Noguchi K Niwa A Matsuo M. Galectin-3: a potential prognostic and diagnostic marker for heart disease and detection of early stage pathology. Biomolecules. (2020) 10:1277. 10.3390/biom10091277 32899694 PMC7565392

[34] Aitken E. Cardiovascular changes occurring with occlusion of a mature arteriovenous fistula. J Vasc Access. (2015) 16:459–66. 10.5301/jva.5000336 25634156

